# The Register of Antarctic Marine Species (RAMS): a ten-year appraisal

**DOI:** 10.3897/zookeys.524.6091

**Published:** 2015-09-30

**Authors:** Quentin Jossart, Camille Moreau, Antonio Agüera, Claude De Broyer, Bruno Danis

**Affiliations:** 1 Université Libre de Bruxelles, 50 avenue Franklin Roosevelt, 1000 Brussels, Belgium; 2 Institut Royal des Sciences Naturelles de Belgique, 29 rue Vautier, 1000 Brussels, Belgium

**Keywords:** Register of Antarctic Marine Species, Biodiversity, Southern Ocean, Antarctic, Bibliometrics, Asteroidea

## Abstract

The Register of Antarctic Marine Species (RAMS) is a marine species database that manages an authoritative taxonomic list of species occurring in the Southern Ocean. RAMS links with several other initiatives managing biogeographic or genomics information. The current paper aims to briefly present RAMS and provides an updated snapshot of its contents, in the form of a DarwinCore checklist (available through http://ipt.biodiversity.aq/resource.do?r=rams) and illustrative barplots. Moreover, this article presents a ten year appraisal (since the creation of RAMS). This appraisal first focuses on RAMS bibliometrics. We observed that RAMS was cited (Google Scholar) in 50 distinct publications among which 32 were peer-reviewed in 18 different journals. Three journals (Antarctic Science, Polar Biology, ZooKeys) represent almost 40% of these peer-review publications. The second appraisal focuses on the evolution of new RAMS records. We observed an important decrease in data additions since 2011. As a case study, we focused on an original dataset for a specific group (Asteroidea, Echinodermata). It appears that around one hundred species of asteroids are lacking in RAMS despite the relatively high availability of these data. This suggests that the users’ community (or collaborative projects such as AquaRES) could be helpful in order to maintain the RAMS database over the long term.

Register of Antarctic Marine Species

## Rationale

The Register of Antarctic Marine Species (RAMS) is one of the regional species databases within the World Register of Marine Species (WoRMS, http://marinespecies.org) (Costello et al. 2013). RAMS compiles and manages an authoritative taxonomic list of species occurring in the Southern Ocean, establishing a dynamic benchmark for marine biodiversity research, conservation and sustainable management (De Broyer and Danis 2011). RAMS serves as a taxonomic reference for biogeographic information systems such as biodiversity.aq (Van de Putte et al. 2015) and iOBIS (Ocean Biogeographic Information System, IOC 2015). It also links with several other initiatives, including GenBank (http://www.ncbi.nlm.nih.gov/genbank/) and Barcode of Life (http://www.barcodeoflife.org/, see also De Broyer and Danis 2011 for additional information on RAMS concept and primary goals).

RAMS is managed by an Editorial Board which includes an Executive Committee and a team of Taxonomic Editors. The RAMS Executive Committee has an advising role in the development of RAMS and proposes Taxonomic Editors to take up responsibility for the maintenance of the database content. These editors are taxonomic experts and are in charge of the content and data quality control of their specific taxon. In March 2015, RAMS was administered by 59 taxonomic experts from 18 countries and 43 universities, museums or institutes.

**Figure 1.**
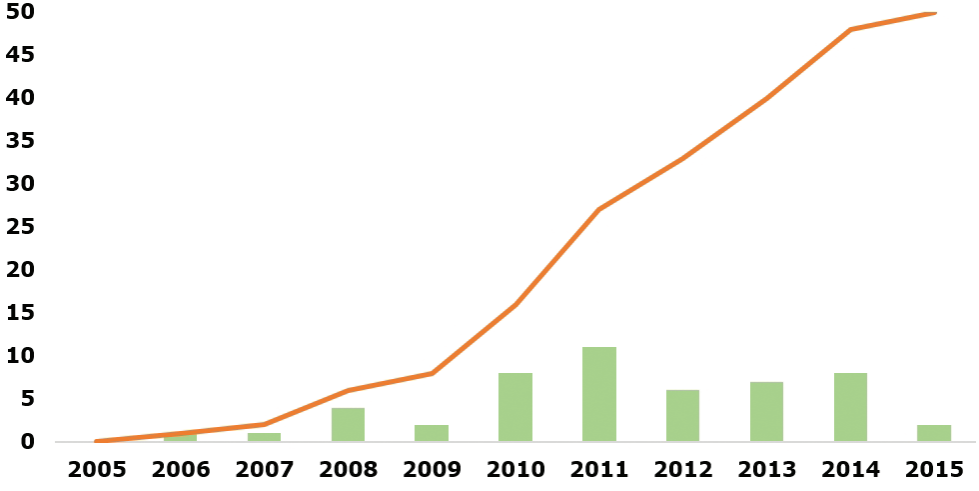
Cumulative number of publications citing “Register of Antarctic Marine Species” since 2005 (orange curve) and number of publications per year citing “Register of Antarctic Marine Species” (green barplot).

**Figure 2.**
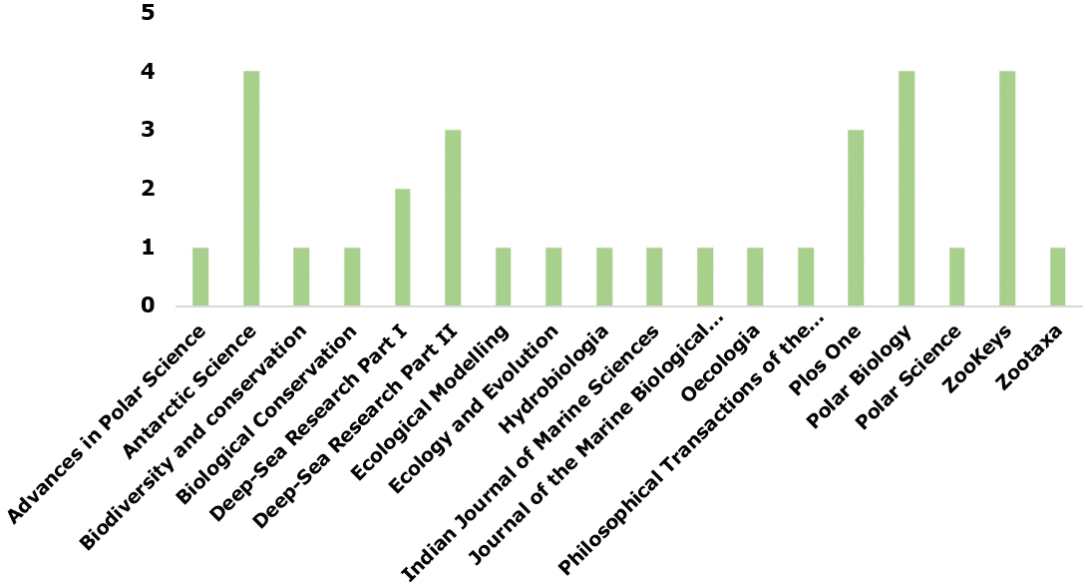
Number of distinct publications citing “Register of Antarctic Marine Species” per peer-reviewed journal.

Since its creation in 2005, the “Register of Antarctic Marine Species” was cited (Google Scholar, https://scholar.google.com) in 50 distinct publications (Fig. 1) that were themselves cited 492 times. Among these 50 publications, 32 were peer-reviewed in 18 different journals (mean impact factor: 2.25±1.25). Three journals (Antarctic Science, Polar Biology, ZooKeys) represent almost 40% of these peer-review publications and this percentage even exceeds 60% if the journals “Deep-sea Research Part I & II” and “Plos One” are added (Fig. 2).

## Taxonomic coverage

General taxonomic coverage description: RAMS checklist (DarwinCore) is available through the GBIF Integrated Publishing Toolkit (http://ipt.biodiversity.aq/resource.do?r=rams).

The taxonomic scope of RAMS covers Antarctic and sub-Antarctic species from three realms (see operational limits in the Spatial coverage section): the sea floor (meio-, macro- and megazoobenthos; micro- and macrophytobenthos), the water column (phytoplankton, zooplankton, nekton) and the sea-ice.

**Figure 3.**
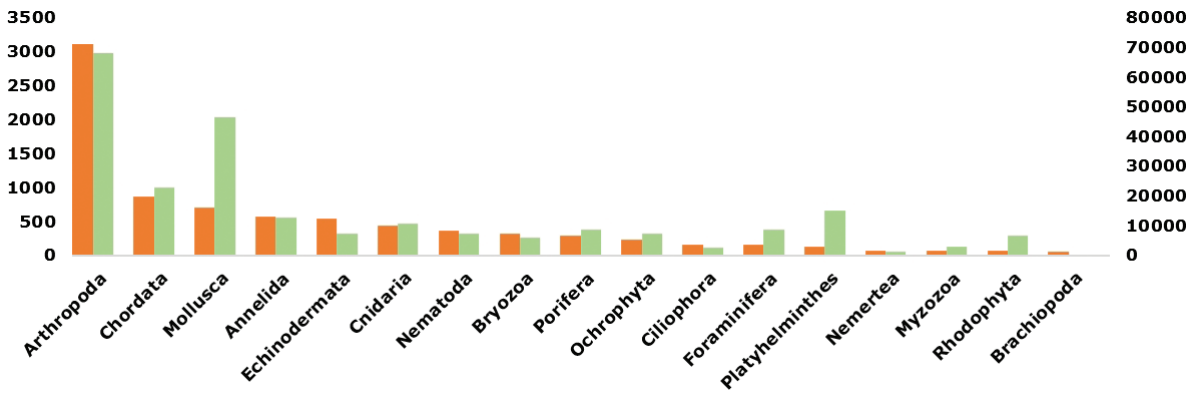
Number of accepted species (marine + non marine) per phylum in RAMS (orange plots, left axis) and in WoRMS (green plots, right axis). Phyla with less than 50 occurrences in RAMS were not represented.

As of March 2015, RAMS includes data on 18,470 taxa and 10,294 species, out of which 81% are taxonomically accepted (see last stats: http://marinespecies.org/rams/aphia.php?p=stats). The percentages of checked taxa (scientific names which have been checked by a taxonomic editor) have a huge variability among phyla (0–100%). Several causes could explain these differences such as editor activity levels or literature accessibility being unequal between groups (e.g. recent literature review vs scattered old publications). Moreover, the uncertain taxonomic status of some groups (e.g. due to new genetic analyses) could also explain some update gaps. Finally, the number of species in a group also greatly influences this percentage (a small group should never reach the highest percentages, even if only one species was not checked).The 8,354 accepted species (8,297 marine vs 57 non-marine) are unequally represented among kingdoms and phyla (Fig. 3). Among kingdoms, Animalia is by far the most represented (7,582) before Chromista (643), Plantae (89), Protozoa (39), Bacteria (1), while Archaea is absent. Regarding phyla, the most abundant, with 3120 accepted species, is Arthropoda followed by Chordata (867), Mollusca (705), Annelida (573) and Echinodermata (537). Interestingly, these five most abundant phyla are the same in WoRMS except for Echinodermata, a fact that highlights again their high specific diversity in the Southern Ocean compared to other oceans (De Broyer et al. 2014).

**Figure 4.**
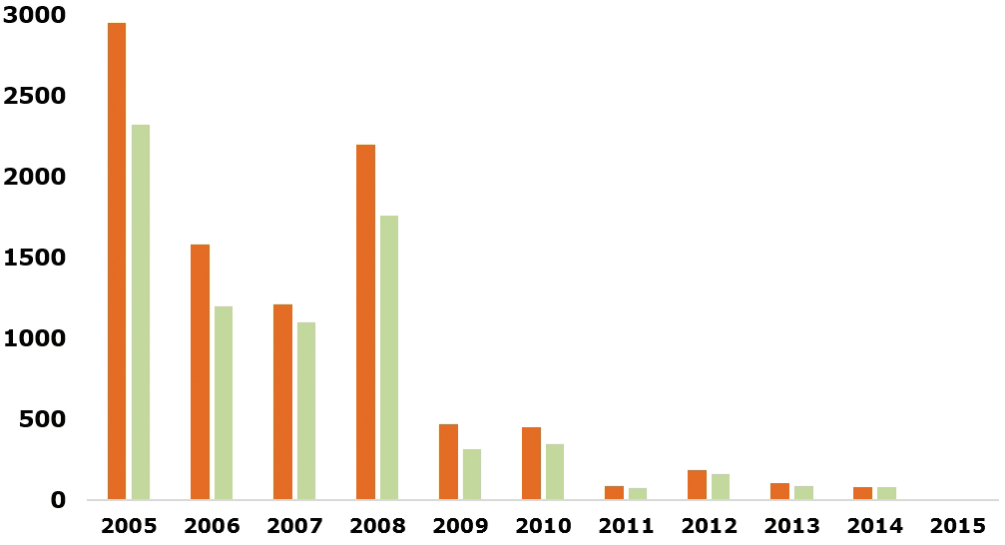
Number of species (left bars in orange) and accepted species (right bars in green) added to RAMS since 2004.

Since 2005, a total of 18,602 taxa (10,547 species) and 15,834 accepted taxa (8,519 accepted species) were added to RAMS, while the great majority of species (87%) and accepted species (88%) were added before 2009 (Fig. 4).

Moreover, for each of the last four years (2011–2014), the numbers of new species and accepted species were always below 200. This suggests that the great majority of available data was already implemented in RAMS and that the last new species are mainly associated to newly-discovered/described taxa.

### A case study: the Asteroidea

**Figure 5.**
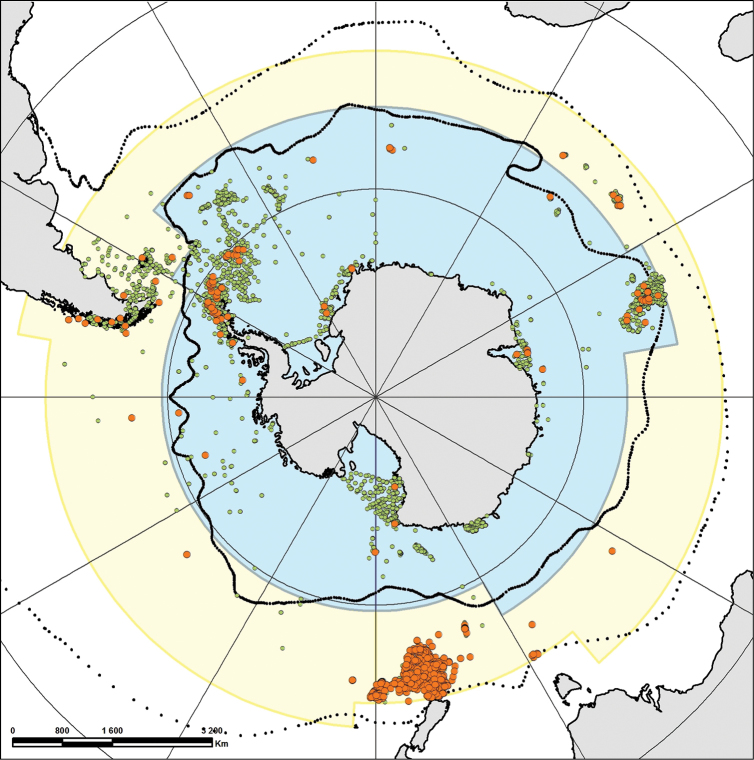
Asteroidea occurrences of RAMS species (green circles) and non-RAMS species (orange circles) within the RAMS area of interest, Antarctic area (light blue) and Sub-Antarctic area (light yellow). Black continuous line is the Polar front and the black dashed line is the Subtropical front.

To illustrate this prediction (“available data are already uploaded in RAMS”), as well as the quality of the RAMS database, we focused on a particular taxonomic group, the Asteroidea Class (Echinodermata), to check for potential mistakes or gaps. The choice of this class is justified by the fact that the Asteroidea are known to be highly diversified in the Southern Ocean (Danis et al. 2014). We built an original database, mining data from iOBIS (2015), the Biogeographic Atlas of the Southern Ocean (Danis et al. 2014) but also in early and recent literature. In this new dataset, around 289 species (13,308 occurrences) were found and only two spelling mistakes in RAMS species names were reported. However, RAMS lacks 98 species (1,160 occurrences) including data on 191 species (12,148 occurrences) (Fig. 5).

## Conclusion

These gaps in Asteroidea data have probably three main causes. First, some recent papers (e.g. Janosik and Halanych 2010), describing new species, have not been taken into account in RAMS since their publication. Secondly, some other species are absent due to a lack of distribution information. Some species are indeed not reported within the RAMS area of interest despite their presence in it. This is especially true for species from New-Zealand where the Campbell Plateau extends far South within the Sub-Antarctic area. However, these species are often reported as New-Zealand species but not Southern Ocean species (e.g. McKnight 2006). Thirdly, other species not referenced in RAMS are linked to early publications (e.g. end of 19^th^ century) that were not digitalized and can be difficult to access.

The case-study of Asteroidea highlights the fact that, despite the great work and expertise of taxonomic editors, some issues can arise even ten years after the creation of RAMS. Indeed, by checking only one class, we found more new entries than those added for the whole RAMS in 2014. Therefore, the user community is encouraged to help the network of taxonomic editors by contacting them (http://marinespecies.org/rams/aphia.php?p=editors) when they detect any mistake or gap during their RAMS utilization. In addition to gathering experts at specific workshops, several initiatives to address existing gaps in RAMS were/are planned. The development of the Biogeographic Atlas of the Southern Ocean (De Broyer et al. 2014) as well as the SCAR Expert Group on Antarctic Biodiversity Informatics (http://www.scar.org/ssg/life-sciences/eg-abi) both illustrate the interest of incorporating taxonomical works into a broader and more stimulating context. Moreover, LifeWatch grants exist specifically to support editors in addressing gaps in their taxonomic group. Finally, RAMS is currently being enhanced with new data-cleaning tools in the framework of the AquaRES project (Aquatic Species Register Exchange and Services: http://odnature.naturalsciences.be/aquares). The main objective of this project is to improve the quality, interoperability and public availability of three major Global Species Directories, namely RAMS, WoRMS and FADA (Freshwater Animal Diversity Assessment, fada.biodiversity.be, Balian et al. 2007) as well as their data exchange with international initiatives (e.g. Encyclopedia of Life – http://eol.org, Ocean Biogeographic Information System – OBIS – http://iobis.org, Global Biodiversity Information Facility – GBIF – http://gbif.org, LifeWatch – http://lifewatch.be). It is expected that these will provide benefits to RAMS in late 2015 – early 2016.

## Spatial coverage

General spatial coverage: The RAMS area of primary interest is the Antarctic area corresponding to the water masses south of the Polar Front extending to the coasts of the Antarctic continent (Fig. 5). It also includes coverage oft he Sub-Antarctic area (waters from the Polar Front to the Subtropical Front, Fig. 5).

Below are the operational limits for RAMS data as presented on the SCAR-MarBIN website (see Fig. 5; more information can be found on the following doc file: http://scarmarbin.be/documents/RAMS_GeoScope.doc)

**ANTARCTIC AREA**:

– True northern limit: Antarctic Polar Front (or Antarctic Convergence, 48°S to 63°S, convenient average limit: 55°S).

– Operational northern limits:

South Atlantic:

– Between 60°W and 50°W: 57°S

– Between 50°W and 30°E: 50°S

Indian Ocean:

– Between 30°E and 80°E: 50°S

– Between 80°E and 150°E: 55°S

South Pacific:

– Between 150°E and 60°W: 60°S

**SUB-ANTARCTIC AREA**:

– True southern limit: the Antarctic Polar Front (48°S to 63°S, convenient average limit: 55°S)

– True northern limit: the northernmost limit of the Southern Ocean s.l., i.e. the northern limit of the extension of the Sub-Antarctic water masses, which corresponds to the (nearly) circumpolar Subtropical Front (30°S to 47°S, convenient average limit: 43°S).

– Operational northern limits for data:

**Table:** 

South Atlantic and Indian Ocean:	- Between 60°W and 140°E: 43°S
Pacific Ocean:	- Between 140°E and 176°W: 48°S
	- Between 176°W and 80°W: 45°S
	- Between 80°W and 72°W: 41°S

Shapefiles for the RAMS Area of Interest can be downloaded at http://share.biodiversity.aq/Atlas/Resources/Geographic_Scope/Shapefiles/

## Dataset description

**Object name**: The Register of Antarctic Marine Species

**Character encoding**: UTF-8

**Format name**: Darwin Core Archive format

**Format version**: 1.1

**Distribution**: http://ipt.biodiversity.aq/resource.do?r=rams

**Publication date of data**: 27/11/2014

**Language**: English

**Metadata language**: English

**Date of metadata creation**: 27/11/2014

**Hierarchy level**: Dataset
